# Mass Spectrometry-Based Proteomics for Seafood Allergen Detection and Quantification: Current Trends and Technological Frontiers

**DOI:** 10.3390/ijms26188962

**Published:** 2025-09-15

**Authors:** Manuel G. Amado, Manuel Pazos, Mónica Carrera

**Affiliations:** Department of Food Technology, Spanish National Research Council (CSIC), Institute of Marine Research (IIM-CSIC), 36208 Vigo, Spain; mpazos@iim.csic.es

**Keywords:** seafood, allergen, mass spectrometry, detection, quantification, food safety, parvalbumin, tropomyosin

## Abstract

Food allergy is a growing global health concern, with seafood representing one of the most significant sources of allergic reactions. The primary allergens responsible for fish and shellfish allergies are β-parvalbumins and tropomyosin, respectively. Therefore, ensuring food safety requires precise and reliable methods for the detection and quantification of these molecules. Traditional approaches, such as ELISA and PCR, have notable limitations in terms of specificity, sensitivity, and multiplexing capabilities. In contrast, liquid chromatography coupled with mass spectrometry (LC-MS) has emerged over the past decade as a powerful alternative, offering enhanced accuracy and analytical depth. Various LC-MS-based strategies have been developed for the identification and quantification of seafood allergens, contributing to improved allergen monitoring and risk assessment. Nevertheless, the routine implementation of these methods in analytical laboratories still faces several challenges, including high equipment costs, complex workflows, and the need for standardized reference materials and protocols. Continued technological advances and validation efforts are necessary to overcome these barriers and to integrate LC-MS-based techniques into routine food allergen testing.

## 1. Introduction

Food allergies are pathological immune reactions triggered by the ingestion of food protein antigens, known as allergens [1,2,3]. In affected individuals, the immune system overreacts to these harmless substances, while those without allergies experience no adverse effects. Exposure to allergenic foods, even in minimal amounts, can trigger an IgE-mediated response, leading to clinical symptoms ranging from gastrointestinal disorders and airway inflammation to life-threatening anaphylactic reactions [1,2,3].

Food allergies are more prevalent in children and tend to decrease with age [1,2,3]. Although accurate epidemiological data are lacking, it is estimated that food allergies affect 6–8% of young children and 3–4% of adults [4], with prevalence appearing to be on the rise [2,3,4,5]. Moreover, there is no cure for food allergy and the only available approach is strict avoidance of allergenic foods. For these reasons, food allergy represents a growing health concern, highlighting the urgent need for effective strategies in prevention, diagnosis, and management.

To ensure consumer safety, several regulations have been implemented [6]; however, some products on the market may contain trace amounts of undeclared allergens due to unintentional cross-contamination during food production. To address this issue, Precautionary Allergen Labeling (PAL) is used to inform consumers of potential risks [7]. Nevertheless, PAL is often counterproductive as it is not based on quantitative risk assessments and may lead allergic individuals to unnecessarily avoid products that do not actually contain the indicated allergen [8]. As a result, affected individuals may be deprived of the nutritional value of certain foods and experience anxiety when purchasing or consuming them. This creates a significant burden, highlighting the need for precise methods to detect and quantify allergens in order to support more accurate and evidence-based labeling.

Among the regulated priority allergens, seafood represents a particularly relevant category and includes various edible marine animals such as fish, crustaceans, and mollusks [9,10]. For culinary reasons, the two invertebrate groups of crustaceans and mollusks are often grouped together as shellfish. While ingestion is the primary trigger for allergic reactions, sensitive individuals may also react to handling seafood or inhaling cooking vapors, especially in domestic and occupational settings [9,10,11]. Fish allergy affects around 1% of the population, although prevalence is higher in coastal countries with high fish consumption and in areas where fish processing is a major industry [1,10,12]. Research indicates that shellfish allergy is generally more prevalent than fish allergy in the overall population, reaching up to 3% [1,10].

To address this challenge effectively, it is essential to study it at the molecular level. The protein β-parvalbumin (β-PVALB) is considered the primary fish allergen, while tropomyosin (TM) is the main allergen in crustaceans [1,9,10]. However, the diversity of allergens present in seafood is substantial as each species may contain multiple distinct allergenic proteins. Additionally, allergens from different species can trigger cross-reactivity in sensitized individuals [1,9,10]. These factors present a major challenge for the comprehensive analysis and characterization of seafood allergens.

To advance the study of these proteins and to support the development of effective prevention and control strategies, proteomics has emerged as a fundamental tool. While traditional detection techniques such as ELISA and PCR have limitations, modern mass spectrometry (MS) offers superior specificity, reproducibility, and the ability to perform multiplexed and parallel analyses of peptides and proteins [13]. Bioinformatic analysis of MS data has driven significant advancements, expanding analytical capabilities and revolutionizing high-throughput protein analysis. Consequently, liquid chromatography coupled with mass spectrometry (LC-MS) has been widely applied to the study and monitoring of allergens [14,15,16].

This review explores MS-based approaches for the detection and quantification of seafood allergens, with a focus on technological advancements, current challenges, and future perspectives to enhance food safety and regulatory compliance.

## 2. Allergens in Seafood

**Figure 1:**
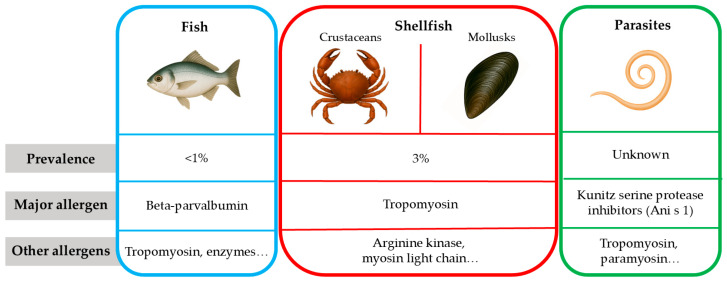
Classification of seafood groups and allergens. Prevalence values have been taken from reference [1].

As previously discussed, seafood allergens are diverse and exhibit cross-reactivity among different species (Figure 1).

### 2.1. Fish Allergens

Parvalbumins (PVALBs), considered the major fish allergen, are low-molecular-weight (10–12.5 kDa) calcium-binding proteins, typically comprising 108–109 amino acids, and are structurally related to calmodulin and troponin C [17,18]. They play a crucial role in calcium regulation in fast-twitch muscle fibers, although they are also present in non-muscle tissues such as the brain, bones, skin, and gonads [17].

PVALBs are responsible for 95% of fish-induced food allergies [17]. The PVALB gene family consists of two major types, α and β, with β further divided into β1 and β2 [17,18]. β-PVALBs have a higher affinity for Ca^2+^ ions compared to α-PVALBs, making them the primary allergens in fish. In contrast, α-PVALBs are generally considered non-allergenic. Cartilaginous fish (e.g., sharks and rays) predominantly express α-PVALBs in their muscle tissue, which correlates with their lower incidence of allergic reactions compared to bony fish [19].

PVALBs are highly resistant to heat, enzymatic digestion, and food processing [9,17]; however, their IgE reactivity has been shown to decrease when tissues are exposed to high temperatures or subjected to various seafood processing methods [20].

A high diversity of PVALB genes has been observed in teleost fish, yet these proteins remain highly conserved, sharing >45% amino acid identity across species [18]. Certain linear and conformational epitopes are particularly well-preserved, serving as common IgE recognition sites, which can lead to cross-reactivity even between distantly related fish species [17,18,21].

Other fish allergens have also been studied. Fish TM, although being less allergenic than PVALBs, is more heat-resistant and might be the main allergen in autoclaved food products, such as canned fish [22,23]. Glycolytic enzymes, including aldolase A and β-enolase, have been identified as allergens; however, their relevance is lower due to their heat and processing sensitivity [1,9]. Additionally, fish collagen, vitellogenin from roe, and other molecules have been proposed as potential fish allergens, although their clinical significance remains under investigation [1,9].

### 2.2. Shellfish Allergens

Tropomyosin (TM) is the major allergen found in all shellfish species (crustaceans and mollusks) [1,9,24]. It belongs to a family of highly conserved actin-binding proteins that play a key role in muscle contraction in both vertebrates and invertebrates. Beyond muscle tissue, TM is also present in the brain, platelets, fibroblasts, and various non-muscle cells [1,9]. Recognized as a pan-allergen, around 60% of shellfish-allergic individuals exhibit IgE reactivity to TM [1]. However, limited information is available regarding the allergenicity of molluscan TM [25].

TM’s secondary and tertiary structure provides it with high structural stability, allowing it to withstand heat and high-pressure processing while retaining its allergenic properties [1,9]. Some food-processing methods, including thermal and pressure treatments, may not only fail to eliminate its allergenicity but can even enhance IgE binding, potentially increasing its immunogenic potential [1].

Due to its highly conserved sequence, with over 95% amino acid identity among prawns, crabs, and lobsters, TM exhibits strong cross-reactivity within crustaceans [1,9,26]. The homology of TM between crustaceans and mollusks is 55–65%, leading to frequent cross-reactivity [1,26,27]. Additionally, TM contributes to cross-reactivity between shellfish and other invertebrates, such as insects, mites, and nematodes, in which it is also highly conserved [1,9,24].

Arginine kinase (AK), although less heat-resistant than tropomyosin, is considered the second most relevant shellfish allergen, with 10–20% of shellfish-allergic patients showing sensitization [1,9,26]. AK has been identified and characterized in various crustaceans and mollusks, including crabs, lobsters, prawns, and octopuses. In addition, several minor allergens have been identified in shellfish, including myosin light chain, sarcoplasmic calcium-binding protein (SCP), troponin, paramyosin, hemocyanin, and various proteins found in different crustacean species [1,9,26].

### 2.3. Seafood Parasites Allergens

In addition to seafood species, allergic reactions to fish-borne parasites, particularly *Anisakis simplex*, have significantly increased over the past decade [9,28]. *Anisakis* is a parasitic nematode that primarily affects marine fish but has also been found in shellfish [1]. Consumption of contaminated seafood can trigger severe allergic reactions, even after the parasite is killed through freezing or cooking, as its allergens remain intact [1,9].

Among the 14 characterized *Anisakis*-derived allergens (Ani s1–s14), TM, paramyosin, and protease inhibitors are notable for their heat and gastrointestinal resistance [9]. The TM family is particularly relevant due to its role in cross-reactivity with other invertebrates, although the prevalence of cross-reactivity between *Anisakis* and shellfish remains difficult to assess due to limited population-based studies. Additionally, recent research has identified occupational sensitization to *Anisakis* among fish-processing workers, highlighting its broader impact beyond consumption-related allergies [28]. Therefore, patients allergic to fish or shellfish should be tested for *Anisakis simplex* and *Ascaris lumbricoides* allergy [1].

### 2.4. Emerging New Allergens by Globalization and Climate Change

Due to globalization of seafood trade, our dietary patterns are shifting toward a wider variety of marine products, including species not traditionally consumed in certain regions [29]. This increased exposure to non-native organisms, such as tropical fish, exotic crustaceans, jellyfish, and novel aquaculture species, has introduced new allergenic proteins into the food chain [30,31]. As a result, populations are at greater risk of sensitization to unfamiliar allergens, including PVALB isoforms and TM variants from invasive or farmed species.

Climate change further contributes to this phenomenon by disrupting the ecosystems and marine species distribution [32]. Rising sea temperatures are enabling the spread of exotic species such as lionfish, pufferfish, and non-native crustaceans into new regions, thereby increasing the risk of exposure to novel and potentially allergenic proteins [33,34]. Moreover, thermal stress on marine organisms can enhance the expression of heat-shock proteins (HSPs) or alter known allergenic proteins, potentially increasing their allergenicity [35].

These changes may explain the increasing prevalence of food allergies, and highlight the urgent need for updated allergen monitoring and risk assessment strategies.

## 3. Mass Spectrometry-Based Methodologies for Allergen Detection and Quantification

**Figure 2:**
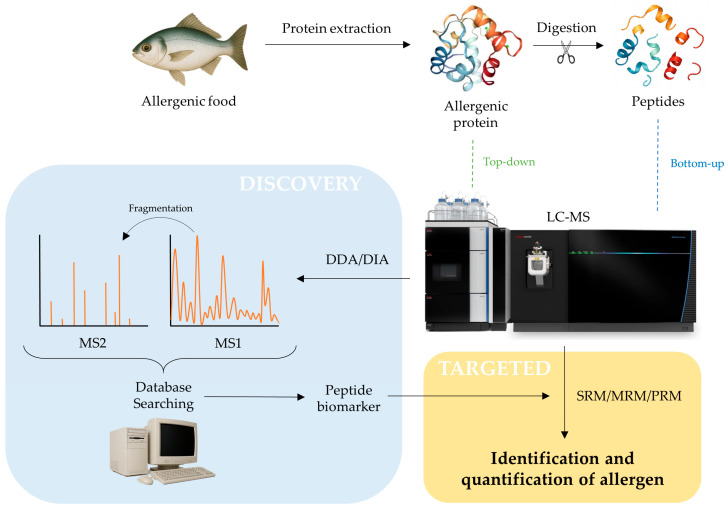
Schematic overview of allergen identification and quantification by LC-MS.

Detecting an allergen in food by LC-MS involves extracting and purifying proteins, separating them via electrophoresis or LC, and identifying potential allergens using MS. The results are analyzed against allergen databases, followed by validation through techniques like ELISA or Western blot. A general workflow for allergen identification and quantification by LC-MS is presented in Figure 2.

### 3.1. Sample Preparation

The biggest challenge in proteomics technology lies in the inherent complexity of cellular proteomes [36]. Seafood samples are often processed and embedded in complex matrices; therefore, proper sample preparation is essential to maximize the isolation of the target protein and minimize contaminants.

Protein extraction typically involves breaking down the tissues into a suspension containing proteins and other biomolecules [36]. In order to extract soluble allergens from seafood muscle tissue, the standard approach consists of mechanical homogenization in an isotonic buffer solution, such as PBS or Tris-HCl [9]. Additionally, some studies have aimed to optimize specific protocols. The addition of SDS (sodium dodecyl sulfate), β-ME (β-mercaptoethanol), and EDTA (ethylenediaminetetraacetic acid) has been shown to increase the extractability of PVALB from mullet and salmon [37]. Enhancing the ionic strength and pH of the extraction buffer can facilitate the solubilization of allergens from oyster tissue [38]. A synergistic effect of buffer additives has been shown to enhance protein extraction from thermally processed shrimp [39]. However, since the allergenicity of the same protein exhibits differences depending on the matrix, the extraction ingredients and conditions must be carefully selected [37].

Protein purification aims to isolate the target protein from complex biological mixtures. Taking advantage of their thermostability, PVALBs and TM are typically purified by heating the sarcoplasmic extracts followed by centrifugation [40]. Immunoaffinity assays are increasingly being employed to further isolate seafood allergens [41].

### 3.2. Liquid Chromatography Coupled to Mass Spectrometry (LC-MS)

So far, ELISA has been the method of choice for allergen detection; however, numerous authors have highlighted its limitations, including false positives, dependence on antibodies, cross-reactivity, and strong susceptibility to matrix effects, among others [42,43]. On the other hand, PCR-based techniques target the gene rather than the protein itself, offering no direct insight into the actual allergen quantity. Currently, MS is emerging as a promising tool for identification, characterization, and quantification of proteins due to its high sensitivity, robustness, and ability to perform high-throughput analysis on a large scale [44,45].

Prior to MS analysis, it is essential to separate the proteins present in the sample to reduce complexity and improve the accuracy of identification [36]. Common techniques include gel electrophoresis (such as SDS-PAGE or 2D-PAGE) and LC, which help isolate proteins or peptides based on their specific characteristics. Gel-based fractionations are commonly followed by MALDI-TOF, while LC separation is typically coupled to MS via an Electrospray Ionization (ESI) interphase [36].

In MS analysis, bottom-up and top-down are two main strategies for protein analysis (Figure 2). The bottom-up approach involves digesting proteins into peptides before LC-MS analysis, allowing for high sensitivity and extensive peptide identification; the top-down approach analyzes intact proteins, preserving structural information such as post-translational modifications (PTMs) and sequence variants. Both workflows have distinct advantages and are chosen based on the specific goals of a proteomics study.

#### 3.2.1. Bottom-Up Approach

Bottom-up is the most commonly used approach for allergen detection and quantification [46,47]. Often referred to as “shotgun proteomics”, it involves digesting the proteins (typically with trypsin) to generate a variety of peptides, which are then separated by LC and analyzed by MS or tandem MS (MS/MS) [13]. Then, the experimentally derived peptide masses or fragment ions are matched to database entries that include in silico theoretical spectra for protein identification. To improve the accuracy of allergen identification, several specialized databases have been developed, including AllergenOnline and Allergome [48]. If the protein sequence is not present in the available databases, de novo sequencing should be performed.

Two sequential proteomics approaches, namely discovery proteomics and targeted proteomics, are conducted [13] (Figure 2). In the early stages of research, discovery techniques are employed to identify and characterize as many proteins as possible in order to uncover biomarkers. It is essential to select peptides that are specific to the target allergen, thermally stable, and analytically robust. To achieve this, two common acquisition methods are data-dependent acquisition (DDA) and data-independent acquisition (DIA) (Table 1) [13]. DDA selects the most intense precursor ions from an initial MS scan for fragmentation in MS/MS, making it effective for identifying high-abundance peptides but potentially missing low-abundance ones. In contrast, DIA systematically fragments all precursor ions within defined mass-to-charge (*m*/*z*) windows, providing a more comprehensive and reproducible peptide coverage. In fact, DIA has gained popularity for quantitative applications due to its higher consistency and reduced sampling bias across complex samples.

Once the biomarkers are discovered, targeted proteomics techniques are performed to accurately detect and quantify them [49]. The main methods used are Selected Reaction Monitoring (SRM), Multiple Reaction Monitoring (MRM), and Parallel Reaction Monitoring (PRM) (Table 1). SRM is typically performed on triple–quadrupole (QqQ) instruments and involves the selection of a specific precursor ion in the first quadrupole; fragmentation in the second (collision cell); and detection of a specific product ion in the third quadrupole [50]. When multiple such transitions are monitored within a single run, the approach is referred to as MRM. In contrast, PRM is performed on high-resolution mass instruments such as Orbitrap or Q-TOFs, where the precursor ion is isolated and all resulting fragment ions are detected in parallel, generating a full MS/MS spectrum [51]. PRM offers greater specificity and post-acquisition flexibility, making it a powerful alternative for targeted peptide quantification and biomarker verification.

**Table 1:** Comparison of common MS-based techniques for allergen detection.

MS Technique	Sensitivity	Complexity	Cost	Best Use Cases	Ref.
DIA	Moderate; biased toward abundant peptides	Moderate; limited MS2 scans performed	Medium	Discovery proteomics; identifying high-abundance allergens	[13]
DDA	High; improved reproducibility across samples	High; advanced data analysis required	Medium–High	Biomarker discovery in complex matrices; quantitative proteomics	[13]
SRM	High; very specific for targeted peptides	Moderate	Medium	Targeted allergen detection; validation of biomarkers	[50]
MRM	High; very specific for targeted peptides	Moderate	Medium	Routine quantification of known allergens in food products	[50]
PRM	Very high; full MS/MS spectrum increases specificity	Medium	High; requires high-resolution MS	Targeted quantification with high specificity; verification of allergenic peptides	[51]
LFQ	Moderate; dependent on instrument stability	Low–Moderate	Low	Large-scale comparative studies; relative quantification without labels	[52]
SILAC	High; accurate relative quantification	High; requires metabolic labeling	High	Model systems, cell culture studies; precise quantitative proteomics	[53]
TMT	Very high; high multiplexing	High; requires chemical labeling	Very high; expensive reagents	Large-scale comparative proteomics; simultaneous analysis of multiple food matrices	[54]
AQUA	Very high; absolute concentration determination	Medium; must be combined with SRM, MRM, or PRM	Medium–High; requires synthetic peptides	Accurate quantification of specific allergens; establishing thresholds (e.g., VITAL levels)	[55]
MS3	Very high; reduces interference in complex samples	Very high	Very high; advanced instrumentation required	Quantification of low-abundance allergens in highly complex food matrices	[56]

#### 3.2.2. Top-Down Approach

Bottom-up proteomics presents significant limitations in the identification of protein isoforms and the modification patterns that generate the diverse proteoforms. In contrast, top-down proteomics, which involves the analysis of intact proteins, provides a more comprehensive approach for assessing allergen proteoforms [57]. This is particularly relevant as different proteoforms of the same allergen may exhibit varying allergenic potentials [58,59]. Despite its advantages, top-down MS requires advanced instrumentation, complex data analysis, and its performance can be limited by ion suppression and signal overlap in complex matrices [57].

Within top-down workflows, ultraviolet photodissociation (UVPD) has emerged as an interesting choice for fragmentation [60]. By using high-energy ultraviolet photons, UVPD induces cleavage at multiple backbone sites, generating a diverse and abundant set of fragment ions. This results in extensive sequence coverage and more detailed structural characterization of allergenic proteins [60,61].

Additionally, an intermediate approach, called middle-down, analyzes large protein fragments generated by limited proteolysis, combining the sequence coverage of bottom-up with the structural insights of top-down.

### 3.3. Quantification via LC-MS

In allergen detection, the amount of allergenic protein is a critical factor. Significant efforts have been made on determining the Minimum Eliciting Doses (MEDs) in food-allergic individuals [62,63]. The VITAL (Voluntary Incidental Trace Allergen Labelling) program, developed by the Allergen Bureau of Australia and New Zealand, is one of the most recognized frameworks for establishing reference doses below which PAL is unnecessary [64,65]. Consequently, analytical methods must be sufficiently sensitive to quantify allergens below the VITAL established thresholds, and MS technology has demonstrated its capability to meet these demands [66]. The most recent review of VITAL threshold levels (VITAL 4.0; 2024) established reference doses expressed in mg_allergen_/kg_food_ as 50 mg/kg for fish, 2000 mg/kg for crustaceans, and 200 mg/kg for mollusks (based on a standard portion size of 100 g), intended to elicit a reaction in no more than 5% of allergic individuals [64].

Protein quantitation by LC-MS can be achieved using two main approaches: label-free and label-based methods, each with distinct advantages and limitations (Table 1) [67]. Label-free quantitation (LFQ) consists of comparing the peak areas or intensities of peptides across a sample set to infer relative protein abundances [52]. LFQ does not require a specific sample preparation, making it suitable for analyzing large numbers of diverse or complex biological samples. Although it can be more susceptible to run-to-run variability, advancements in instrumentation and software have significantly improved its accuracy and reproducibility.

In contrast, label-based approaches involve more extensive sample preparation but offer improved accuracy and reproducibility [67]. Stable Isotope–Labeled techniques (SILAC) involve incorporating heavy isotopes (e.g., ^13^C or ^15^N) into proteins during cell growth [53]. Labeled (heavy) and unlabeled (light) samples are mixed, digested, and analyzed by LC-MS, enabling accurate relative quantification based on mass differences. Another strategy, called dimethyl labeling, modifies peptides with light, medium, or heavy formaldehyde, providing a simple and efficient method for relative quantification [68]. Isobaric Tags for Relative and Absolute Quantification (iTRAQ) and Tandem Mass Tag (TMT) labeling use isobaric tags that bind to peptides and release distinct reporter ions upon MS2 fragmentation, enabling simultaneous protein identification and relative quantification across multiple samples in one LC-MS/MS run [54,69].

While these techniques provide information on relative protein abundance, several methods enable absolute quantification. One common method is AQUA (Absolute QUAntification), based on the use of internal standards or Stable Isotope–Labeled peptides (SILs), which are spiked into samples prior to analysis [55]. The known concentration of these standards allows for the direct calculation of the protein concentration in the sample. Recently, concatenated SIL peptides, created by linking multiple target peptides into a single labeled construct, have been used for multiplexed analysis [70]. Another approach involves using calibration curves, where known concentrations of proteins are analyzed alongside the samples, providing a reference to quantify the target proteins [71]. Absolute quantification is essential in allergen detection to determine whether a protein is present at levels that may pose a risk to sensitive individuals.

However, quantifying low-abundance proteins in complex samples remains a significant challenge. Innovations such as MS3, which adds a third-stage fragmentation to improve identification, may enhance specificity and reduce matrix interference, enabling detection of trace allergen amounts [56]. Similarly, ion-mobility mass spectrometry provides an orthogonal separation based on ion shape and size, further increasing resolution and mitigating matrix effects [72].

### 3.4. System Biology and Machine Learning

Recent advances in proteomics are reshaping the field of allergen detection. Systems biology approaches integrate proteomics with genomics, transcriptomics, metabolomics, and lipidomics, enabling a more comprehensive understanding of allergen expression, regulation, and interaction within the food matrix [73]. Imaging MS techniques, such as MALDI imaging, have been incorporated into proteomic workflows, allowing direct spatial localization of proteins and peptides in tissues or food samples [74]. Additionally, software tools like STRING [75] and Cytoscape [76] facilitate the visualization of protein–protein interaction networks, offering a more holistic view of the underlying biological systems.

Artificial intelligence (AI) and machine learning further advance allergen detection by optimizing data analysis in shotgun proteomics [77]. These technologies improve peptide identification, spectral prediction, and quantification accuracy. Deep learning models like Prosit, especially when combined with data-independent acquisition (DIA), enable high-throughput analysis and robust biomarker discovery [78]. Similarly, Chimerys™ (Thermo Fisher Scientific) is an AI-powered search engine for mass spectrometry data that enhances PTM identification through open search capabilities, allowing for the detection of unexpected or rare modifications with high confidence [79]. As proteomic datasets grow, these tools will become increasingly valuable for automating interpretation and enhancing diagnostic accuracy.

## 4. Applications in Seafood Allergen Detection and Quantification

By applying the aforementioned approaches, numerous methods have been developed to detect and quantify seafood allergens across a wide range of food matrices (Table 2).

### 4.1. Fish

Detection methods in fish primarily focus on β-PVALB as it is the main allergen. A rapid detection method for β-PVALBs in foodstuffs was developed by our group using a fast targeted proteomics scanning approach [80]. The process involves heat-based purification, high-intensity focused ultrasound (HIFU)-assisted digestion, and Selected MS/MS Ion Monitoring (SMIM) to target 19 selected peptides in a Linear Ion Trap (LIT) mass spectrometer. This method enables detection in less than 2 h, even in precooked and processed foods, highlighting its suitability for routine analysis. A simple and sensitive MRM method was developed and validated for the quantification of β-PVALBs, achieving limits of quantification (LOQ) as low as 0.10 µg_PVALB_/g_food_ in flounder (*Paralichthys olivaceus*) [81]. Although MRM3 was also tested, it was ultimately discarded due to insufficient sensitivity. In a different study, a shotgun proteomics approach was employed to characterize and compare the muscle proteome of farmed and wild gilthead sea bream, revealing a higher expression of PVALB in farmed fish samples [82]. The authors compared two quantification approaches, with label-free proving more effective than dimethyl labeling in this context. Not only bottom-up approaches are employed; an efficient top-down method for detecting β-PVALBs has also been developed, utilizing the high resolution of an Orbitrap combined with UVPD [61]. This strategy offers several advantages, including minimal sample preparation, high sensitivity, and extensive protein sequence coverage. Beyond muscle tissue, MALDI-TOF MS has also enabled the identification of two major PVALB isoforms in carp seminal plasma [83]. Furthermore, researchers used an Orbitrap to analyze 26 commercial fish allergen extracts and found significant variability in protein content, allergen composition, and IgE reactivity, highlighting inconsistencies that may compromise the reliability of skin prick tests for fish allergy diagnosis [84].

Many studies focus on characterizing and sequencing PVALBS to facilitate their detection in future analyses. To deepen the understanding of β-PVALBS, Liu et al. implemented a multi-omics strategy incorporating MS-based proteomics, which enabled detailed epitope mapping, cross-reactivity assessment, and evaluation of IgE-binding properties [85]. Complementing these efforts, an advanced proteomics workflow enabled the de novo MS sequencing of 25 novel PVALB isoforms from *Merlucciidae* species, which were subsequently registered in the UniProtKB and Allergome databases (accession numbers: P86739–P86775) [86]. Another top-down proteomics approach employing MALDI-TOF-TOF enabled the complete and unambiguous sequencing of four PVALB isoforms from farmed rainbow trout (*Oncorhynchus mykiss*), allowing the identification of point mutations [87]. Nonetheless, PTMs remain unexplored, despite their potential to modulate allergenicity. In fish PVALBs, alpha-N-terminal acetylation is the only PTM identified to date, as detected in purified Gad m 1 [104] and 25 isoforms from *Merlucciidae* species [86]. In several fish tropomyosins, mass differences observed by MALDI-TOF MS suggest the presence of unidentified PTMs [105].

Although β-PVALBs are the main allergen, identifying novel allergens is essential to broaden our understanding of fish allergen profiles. A widely used approach is immunomagnetic separation (IMS), which employs magnetic beads functionalized with antibodies derived from patient sera to selectively capture potential allergens, followed by their elution and identification via MS. Using this method, researchers discovered a previously unreported allergen in sablefish, named SVBP (small vasohibin-binding protein) [88]. Similarly, Zhao et al. applied this strategy to boiled fish bones and identified 25 potential allergens, of which only two had been previously recognized in the literature [89]. In another study, eight allergens were identified in the exudate of large yellow croaker, including PVALB, histone H4, and cytochrome c, further highlighting the complexity and diversity of fish allergenicity [90].

Moreover, efforts have been made to analyze the effects of food processing and cooking on fish allergenicity [106]. It has been shown that heat treatment can alter the allergenicity of seafood proteins, sometimes reducing it through protein denaturation or degradation but in other cases potentially increasing it by exposing hidden epitopes or generating new allergenic structures [107]. Using 2-DE and MALDI-TOF MS, this study identified PVALB and TM as the main heat-stable proteins in cod [22]. However, TM exhibited greater resistance to autoclaving than PVALB, suggesting it may be the predominant allergen in autoclaved fish products such as canned fish. Additionally, the same study identified secondary allergens, including myosin light chain, myosin heavy chain, triosephosphate isomerase, and troponin I, as heat-stable proteins. Supporting these findings, Taki et al. demonstrated a significant decrease in PVALB content in canned fish compared to conventionally cooked fish [108]. Similarly, another recent MS-based study indicated that both boiling and simulated gastrointestinal digestion further reduce PVALB levels [109]. Therefore, it is conceivable that heat treatments could be employed to reduce the allergenicity of PVALBs in fish products [20,106]. However, non-thermal processing methods should also be explored as alternative strategies. A bottom-up approach revealed that high-pressure treatment (≥430 MPa) significantly degraded several fish muscle proteins, including phosphoglycerate mutase-1, enolase, and creatine kinase, while increasing the abundance of β-PVALB, TM and glyceraldehyde-3-phosphate dehydrogenase [91].

### 4.2. Shellfish

Just like in fish, most detection methods in shellfish focus on the major allergen, which in shellfish is TM. A recent study developed a SILAC-based method for the absolute quantification of TM in complex food matrices, achieving LOQs as low as 1 μg/g in processed foods and sauces [92]. The authors highlight the advantages of the SILAC technique, arguing that introducing internal standards early in the workflow ensures more reliable results as they undergo the same processing steps as the analytes (protein extraction, digestion, etc.), thereby correcting potential errors associated with procedural variations. An AQUA-based method was developed and validated for quantifying TM in foods, enabling the first comparative analysis of TM levels across seven Taiwanese shrimp species and demonstrating that common shrimp (*Litopenaeus vannamei*) has the highest concentration [93]. Another targeted MRM-based method was developed for the accurate detection of crustacean TM in processed foods using shared peptide markers, achieving a limit of detection (LOD) of 0.15 mg_TM_/kg_food_ and an LOQ of 0.5 mg_TM_/kg_food_ [94]. The authors emphasized the importance of evaluating matrix effects and the need for SIL peptides to effectively compensate for them. A comparable approach employed immunoaffinity purification to enhance MS analysis, achieving a slightly improved LOQ of 0.1 mg_TM_/kg_food_ in shrimp and crab [41].

However, there are alternative quantification strategies that do not rely on labeled peptides. One such method, based on the addition of standard peptides and UHPLC–MS/MS, was developed for the simultaneous detection of shrimp and soy allergens in sauce products, achieving LOQs ranging from 0.25 to 5 μg_TM_/g_sauce_ [95]. This approach demonstrated high sensitivity and precision while significantly reducing reagent costs and sample preparation time compared to traditional isotope-labeled methods. In a different study, Korte et al. developed a sensitive and specific MRM-based method for detecting shrimp and lobster allergens in food, capable of identifying trace contaminations down to 1000 μg/g, with MRM3 showing an increase in sensitivity up to 25 μg/g [96].

In order to uncover additional allergenic proteins, a shotgun proteomics workflow combined with bioinformatic tools was used to characterize potential allergens in powdered krill and whiteleg shrimp [97]. Allergen levels were higher in processed samples compared to fresh ones, and eleven common potential allergens were identified. Similarly, a combination of immunoblotting and shotgun MS revealed the presence of 24 novel proteins in Pacific oyster reactive to sera from shellfish-allergic patients [98].

Regarding the effects of food processing on shellfish, it is well-established that TM exhibits considerable resistance to heat [24,110]. The study on Pacific oyster combined immunoblotting and MS, revealing that TM remained detectable after heat treatment, whereas glyceraldehyde-3-phosphate dehydrogenase, fructose bisphosphate aldolase, and AK were only detected in the raw samples [99]. Another study demonstrated that high temperature–pressure treatment reduced the allergenicity of TM in crab (*Scylla paramamosain*) by disrupting its primary, secondary, and tertiary structures [100]. Using LC-MS, the authors analyzed the amino acid sequence and identified seven critical residues involved in epitope formation, whose substitution may reduce allergenicity. These findings may be applied to the development of hypoallergenic variants of TM [111].

### 4.3. Anisakids

Few MS-based methods are currently available for the detection of *anisakids* allergens. In our group, we developed a rapid PRM-based method targeting four peptides from Ani s 9, a known allergenic protein, which enables accurate detection in less than 2 h [101]. In a different study, both LFQ- and AQUA-based methods were developed for the accurate quantification of hemoglobin (Ani s 13) and SXP/RAL-2 protein (Ani s 8) [102]. LFQ, although more versatile since it does not rely on SIL peptides, yielded an LOD of 2 µg/mL, whereas AQUA achieved a lower LOD of 0.1 µg/mL. The same group characterized 13 potential allergens using sera from *Anisakis simplex*-sensitized patients and MS data, thereby expanding the pool of potential biomarkers for detection [103].

## 5. Current Challenges and Future Directions

MS-based proteomics has growing potential for real-world applications. In the food industry, it can complement or even replace assays such as ELISA by enabling multiplexed and highly specific detection of trace allergens in complex products. In clinical settings, MS can support precise diagnosis by identifying sensitizing proteins and monitoring patient exposure. Moreover, regulatory agencies are increasingly considering MS as a reference method for allergen detection and quantification, providing a robust tool to enforce labeling requirements and ensure consumer safety. Nonetheless, despite significant advances in recent decades, key challenges remain for its widespread implementation.

### 5.1. Limitations of MS and Emerging Technologies

Although MS offers numerous advantages over ELISA or PCR and has even been regarded by some authors as the gold standard for allergen detection, it also presents certain limitations that may complicate its routine application. Major drawbacks include high equipment costs, the need for trained personnel, time-consuming protocols, and the relatively demanding nature of sample preparation.

To overcome these challenges, several alternative approaches have been proposed. Ambient mass spectrometry (AMS) enables direct sampling at atmospheric pressure without chromatographic separation, making it well-suited for food analysis; however, its sensitivity for allergen detection has yet to be fully established [112]. Pyrolysis (Py) coupled with gas chromatography (GC) provides an alternative approach to streamline sample preparation by enabling rapid volatilization and introduction of food matrices into the MS instrument [112]. MALDI-MS also contributes to a faster workflow by eliminating the need for LC. Similarly, top-down strategies can further accelerate the process by analyzing intact proteins, thus bypassing the digestion step.

Recent technological advances are enhancing the capabilities of MS. Faster mass spectrometers from Bruker, Thermo, and SCIEX, along with rapid HPLC systems from EvoSep, have greatly increased analysis speed and throughput. For instance, Parallel Accumulation–Serial Fragmentation (PASEF) implemented in Trapped-Ion Mobility Spectrometry–TOF (timsTOF) significantly increases MS/MS acquisition speed and sensitivity, enabling multiplexed and precise detection of trace allergenic proteins in complex food matrices [113]. Furthermore, the Evosep One HPLC system streamlines LC-MS workflows by using pre-packed Evotips and rapid gradients, enabling high-throughput, reproducible proteomic analyses with minimal sample handling [114]. In parallel, there is ongoing progress in the development of miniaturized MS platforms, which hold the potential to bypass laboratory workflows for on site allergen detection [115]. Nevertheless, continued technological progress and methodological refinement are expected in the coming years, paving the way for MS approaches to become reliable and efficient tools in routine allergen detection.

### 5.2. Need for Harmonization

The field still faces significant challenges due to the lack of harmonization across regulatory frameworks and analytical practices [116]. One of the most pressing issues is the absence of officially established threshold levels for many allergens, which complicates risk assessment and accurate labeling. Even when initiatives like the VITAL panel have proposed reference doses, binding national and international regulations remain necessary.

On the other hand, the lack of universally accepted reference materials has been repeatedly emphasized [116,117]. Although AOAC SMPR^®^ (Standard Method Performance Requirements; 2016.002) recommends the use of validated reference materials from sources such as the NIST (National Institute of Standards and Technology) or LGC Standards, the unavailability of standardized materials for certain allergens, along with their inconsistent use in routine laboratory practice, remains a significant challenge [118]. This lack of standardization limits inter-laboratory comparability and method validation.

Moreover, standardizing MS methods for a wide variety of allergens would be highly valuable. Some efforts have been made to detect multiple food allergens in a single analysis [119,120], although to our knowledge, none have specifically targeted seafood allergens. However, given the diverse physicochemical properties of target allergens, the complexity of food matrices, and the variability of processing conditions, a universal sample preparation protocol is unlikely to be feasible [117]. Instead, it may be possible to design specific workflows for groups of food products with similar characteristics, facilitating the validation of multi-allergen analyses.

### 5.3. Research Gaps

Despite growing interest in seafood allergen detection, several important research gaps remain. Most existing studies tend to focus on a narrow range of species, leaving many commonly consumed or regionally important fish and shellfish insufficiently characterized. This lack of coverage is even more pronounced in the case of *Anisakis* spp., whose allergenicity has only gained scientific attention in the past decade despite its relevance in sensitized populations. Furthermore, the cross-reactivity between allergens from different seafood groups, which is essential for accurate diagnosis and risk assessment in allergic individuals, is poorly understood.

A major technical barrier is the limited availability of annotated protein sequences for seafood allergens in public databases. This scarcity limits the development of targeted proteomic methods and complicates the accurate identification and characterization of potential allergens. In parallel, the role of PTMs in seafood allergens remains largely unexplored. While PVALBs appear to exhibit minimal modification, TM has been shown to undergo several PTMs [121,122], although their functional and immunological significance has yet to be elucidated.

Addressing these gaps through comprehensive molecular characterization, improved database curation, and broader species coverage will be essential to advance allergen detection and risk assessment in seafood. With these enhancements, mass spectrometry can be more effectively applied to downstream applications, including the development of biosensors and the creation of hypoallergenic proteins.

### 5.4. Biosensors for On Site Allergen Detection

Biosensors have emerged as a promising tool for the rapid, on site detection of food allergens, offering advantages such as portability, low cost, and minimal sample preparation [123]. Common types include the following: immunosensors, which rely on antibody-antigen interactions; aptasensors, which use nucleic acid aptamers for molecular recognition; and electrochemical sensors, which generate a measurable signal upon allergen binding. A wide range of biosensors have been developed to detect seafood allergens, including those from fish [124,125] and shellfish [126,127], or even both simultaneously [128].

While these platforms show great potential, their reliability depends heavily on the selection of highly specific biomarkers and accurate calibration. In this context, MS technology plays a crucial role, not only in the initial identification and selection of allergenic biomarkers but also in the validation and standardization of biosensor performance. Therefore, MS can be employed to confirm allergen identity, quantify analyte concentrations for calibration curves, and detect potential cross-reactivity or degradation products, ensuring the biosensor’s specificity and sensitivity in complex food matrices.

### 5.5. Hypoallergenic Proteins

The development of hypoallergenic proteins represents a promising strategy to reduce the risk of allergic reactions while preserving the nutritional and functional qualities of food. In addition to their application in safer food products, these engineered proteins may also serve as therapeutic agents to reduce patient sensitization through immunotherapy [129]. This approach typically involves the identification and targeted modification of IgE-binding epitopes to lower allergenicity without compromising protein stability. Regarding seafood, several studies have been conducted to design hypoallergenic PVALB [130,131] and TM [132,133]. MS plays a key role in this process by enabling precise epitope mapping, monitoring structural integrity, detecting PTMs, and evaluating the stability of modified proteins under food processing conditions.

## 6. Conclusions

LC-MS has significantly advanced the detection and quantification of seafood allergens, offering superior specificity, sensitivity, and multiplexing capabilities compared to traditional methods. These technologies have enabled the development of sophisticated methodologies for the precise detection and quantification of major seafood allergens, as well as their characterization, sequencing, and structural analysis.

Despite these advances, several challenges remain before such approaches can be routinely implemented in analytical laboratories. These include the need for more accessible methodologies, standardized reference materials, and broader coverage of seafood species. Continued research using MS-based tools will support the development of innovative solutions such as biosensors and hypoallergenic protein variants, helping to overcome current limitations in seafood allergy detection and management. In this sense, the next steps will involve investigating seafood allergen PTMs, assessing their potential cross-reactivity among species, and expanding the available database.

## Figures and Tables

**Table 2:** MS methods for allergen detection and quantification.

Method	Biomarker	Performance	Application	Ref.
Fish				
PRM targeting 19 selected peptides in a LIT	β-PVALB	Detection time < 2 h	Rapid detection of β-PVALBs	[80]
MRM/AQUA quantification in a QTRAP	β-PVALB	LOD = 0.02–0.04 µg/g LOQ = 0.07–0.12 µg/g	Absolute quantification of β-PVALB	[81]
Shotgun proteomics approach comparing LFQ and dimethyl labeling in an Orbitrap	-	-	Compare the muscle proteome of farmed and wild gilthead sea bream	[82]
Top-down approach using UVPD in an Orbitrap	β-PVALB	-	Detection of intact β-PVALB	[61]
SDS-PAGE separation and MALDI-TOF MS	-	-	Detect substances of 5–15 kDa in carp seminal plasma	[83]
DDA in an Orbitrap followed by SDS-PAGE and immunoblotting	-	-	Analyze commercial fish extracts for the presence and concentration of fish proteins	[84]
Multi-omics approach combining HPLC-HRMS, genomics, and immunoinformatics	β-PVALB	-	Characterization, epitope confirmation, and cross-reactivity analysis of β-PVALB	[85]
Mass determination by FTICR-MS of intact proteins and SMIM of peptide mass gaps	β-PVALB	-	Complete de novo sequencing of 25 new β-PVALB isoforms	[86]
Top-down proteomics in a MALDI-TOF	β-PVALB	-	Sequencing of four PVALB isoforms from farmed rainbow trout	[87]
IMS followed by MS analysis	-	-	Identification of novel fish allergens	[88,89,90]
2-DE and MALDI-TOF	-	-	Identification of heat-stable proteins in cod	[22]
2-DE and bottom-up analysis in an Orbitrap	-	-	Detection of protein abundance variations after high-pressure treatment	[91]
Shellfish				
SILAC-based method using UPLC-MS	TM	LOD = 0.5–5 µg/g LOQ = 1–10 µg/g	Absolute quantification of TM in complex food matrices	[92]
AQUA-based method in an IT	TM	LOD = 0.072 ng/μL LOQ = 0.219 ng/μl	Comparison of TM levels in seven shrimp species	[93]
MRM using shared peptide markers	TM	LOD = 0.15 µg/g LOQ = 0.5 µg/g	Absolute quantification of TM	[94]
Immunoaffinity purification and MRM in a QqQ	TM	LOQ = 0.1 µg/g	Determination of TM in shrimp and crab	[41]
Standard addition method (label-free) in a QqQ	TM	LOQ = 0.25–5 µg/g	Cost-effective detection of shrimp in sauce	[95]
MRM3 in a QTRAP	Eight shellfish allergens	LOD = 25 µg/g	Sensitive detection of lobster and shrimp allergens in food samples	[96]
Shotgun proteomics combined with bioinformatic tools	-	-	Characterize potential allergens in powdered krill and whiteleg shrimp	[97]
Immunoblotting combined with shotgun proteomics	-	-	Identify novel allergens in Pacific oyster	[98]
SDS-PAGE, immunoblotting, and MS identification in an IT	-	-	Evaluate heat treatment on shellfish allergens	[99]
Primary structure determination in a QqQ using bioinformatic tools	-	-	Identification of critical amino acids in crab TM epitopes	[100]
Anisakids				
PRM method targeting four peptides in an LTQ	Ani s 9	Detection time < 2 h	Rapid detection of Anisakids	[101]
LFQ in a QqQ	Ani s 13 Ani s 8	LOD = 2 µg/mL	Semi-quantitative detection of Anisakids	[102]
AQUA in a QqQ	Ani s 13 Ani s 8	LOD = 0.1 µg/mL	Absolute quantitative detection of Anisakids	[102]
SDS-PAGE of patient serum and protein identification by nLC/QqQ	-	-	Identification of novel allergens in Anisakids	[103]

## Abbreviations

The following abbreviations are used in this manuscript:

2-DE	Two-dimensional Gel Electrophoresis
AI	Artificial Intelligence
AK	Arginine kinase
AQUA	Absolute quantification
DDA	Data-dependent acquisition
DIA	Data-independent acquisition
ELISA	Enzyme-Linked Immunosorbent Assay (ELISA)
ESI	Electrospray Ionization
HIFU	High-intensity focused ultrasound
HSP	Heat-shock protein
IMS	Immunomagnetic separation
IT	Ion Trap
iTRAQ	Isobaric Tags for Relative and Absolute Quantification
LC-MS	Liquid Chromatography coupled to Mass Spectrometry
LFQ	Label-free quantitation
LIT	Linear Ion Trap
LOD	Limit of detection
LOQ	Limit of Quantitation
MALDI-TOF	Matrix-Assisted Laser Desorption/Ionization–Time of Flight
MRM	Multiple Reaction Monitoring
PAL	Precautionary Allergen Labeling
PASEF	Parallel Accumulation–Serial Fragmentation
PCR	Polymerase Chain Reaction
PRM	Parallel Reaction Monitoring
PTM	Post-translational modification
QqQ	Triple–quadrupole
SCP	Sarcoplasmic calcium-binding protein
SIL	Stable Isotope–Labeled peptide
SRM	Single Reaction Monitoring
timsTOF	Trapped-Ion Mobility Spectrometry–TOF
TM	Tropomyosin
TMT	Tandem Mass Tag
UVPD	Ultraviolet photodissociation
VITAL	Voluntary Incidental Trace Allergen Labelling
β-PVALB	Beta-parvalbumin

## References

[1] Dramburg S., Hilger C., Santos A.F., De Las Vecillas L., Aalberse R.C., Acevedo N., Aglas L., Altmann F., Arruda K.L., Asero R. (2023). EAACI Molecular Allergology User’s Guide 2.0. Pediatr. Allergy Immunol..

[2] Peters R.L., Krawiec M., Koplin J.J., Santos A.F. (2021). Update on Food Allergy. Pediatr. Allergy Immunol..

[3] Sicherer S.H., Sampson H.A. (2018). Food Allergy: A Review and Update on Epidemiology, Pathogenesis, Diagnosis, Prevention, and Management. J. Allergy Clin. Immunol..

[4] Iweala O.I., Choudhary S.K., Commins S.P. (2018). Food Allergy. Curr. Gastroenterol. Rep..

[5] Tang M.L.K., Mullins R.J. (2017). Food Allergy: Is Prevalence Increasing?. Intern. Med. J..

[6] Chang F., Eng L., Chang C. (2023). Food Allergy Labeling Laws: International Guidelines for Residents and Travelers. Clin. Rev. Allergy Immunol..

[7] Bugyi Z., Muskovics G., Tömösközi S. (2023). Rethinking Precautionary Allergen Labelling—Threshold Doses, Risk Assessment Approaches and Analytical Implications. Acta Aliment..

[8] DunnGalvin A., Roberts G., Regent L., Austin M., Kenna F., Schnadt S., Sanchez A., Hernandez P., Hjorth B., Fernandez M. (2019). Understanding How Consumers with Food Allergies Make Decisions Based on Precautionary Labelling. Clin. Exp. Allergy.

[9] Ruethers T., Taki A.C., Johnston E.B., Nugraha R., Le T.T.K., Kalic T., McLean T.R., Kamath S.D., Lopata A.L. (2018). Seafood Allergy: A Comprehensive Review of Fish and Shellfish Allergens. Mol. Immunol..

[10] Davis C.M., Gupta R.S., Aktas O.N., Diaz V., Kamath S.D., Lopata A.L. (2020). Clinical Management of Seafood Allergy. J. Allergy Clin. Immunol. Pr..

[11] Bonlokke J.H., Bang B., Aasmoe L., Rahman A.M.A., Syron L.N., Andersson E., Dahlman-Höglund A., Lopata A.L., Jeebhay M. (2019). Exposures and Health Effects of Bioaerosols in Seafood Processing Workers—A Position Statement. J. Agromedicine.

[12] Moonesinghe H., Mackenzie H., Venter C., Kilburn S., Turner P., Weir K., Dean T. (2016). Prevalence of Fish and Shellfish Allergy: A Systematic Review. Ann. Allergy Asthma Immunol..

[13] Aebersold R., Mann M. (2016). Mass-Spectrometric Exploration of Proteome Structure and Function. Nature.

[14] Marzano V., Tilocca B., Fiocchi A.G., Vernocchi P., Levi Mortera S., Urbani A., Roncada P., Putignani L. (2020). Perusal of Food Allergens Analysis by Mass Spectrometry-Based Proteomics. J. Proteom..

[15] Carrera M., Pazos M., Gasset M. (2020). Proteomics-Based Methodologies for the Detection and Quantification of Seafood Allergens. Foods.

[16] Korte R., Oberleitner D., Brockmeyer J. (2019). Determination of Food Allergens by LC-MS: Impacts of Sample Preparation, Food Matrix, and Thermal Processing on Peptide Detectability and Quantification. J. Proteom..

[17] Mukherjee S., Horka P., Zdenkova K., Cermakova E. (2023). Parvalbumin: A Major Fish Allergen and a Forensically Relevant Marker. Genes.

[18] Dijkstra J.M., Kuehn A., Sugihara E., Kondo Y. (2024). Exploring Fish Parvalbumins through Allergen Names and Gene Identities. Genes.

[19] Stephen J.N., Sharp M.F., Ruethers T., Taki A., Campbell D.E., Lopata A.L. (2017). Allergenicity of Bony and Cartilaginous Fish—Molecular and Immunological Properties. Clin. Exp. Allergy.

[20] Kubota H., Kobayashi A., Kobayashi Y., Shiomi K., Hamada-Sato N. (2016). Reduction in IgE Reactivity of Pacific Mackerel Parvalbumin by Heat Treatment. Food Chem..

[21] Pérez-Tavarez R., Carrera M., Pedrosa M., Quirce S., Rodríguez-Pérez R., Gasset M. (2019). Reconstruction of Fish Allergenicity from the Content and Structural Traits of the Component β-Parvalbumin Isoforms. Sci. Rep..

[22] Tsai C.-L., Perng K., Hou Y.-C., Shen C.-J., Chen I.-N., Chen Y.-T. (2023). Effect of Species, Muscle Location, Food Processing and Refrigerated Storage on the Fish Allergens, Tropomyosin and Parvalbumin. Food Chem..

[23] Ruethers T., Kamath S., Taki A., Le T., Karnaneedi S., Nugraha R., Cao T., Nie S., Williamson N., Mehr S. (2020). Tropomyosin Is A Novel Major Fish Allergen Of Unrecognized Importance. J. Allergy Clin. Immunol..

[24] Cheng J., Wang H., Sun D. (2022). An Overview of Tropomyosin as an Important Seafood Allergen: Structure, Cross-Reactivity, Epitopes, Allergenicity, and Processing Modifications. Compr. Rev. Food Sci. Food Saf..

[25] Emoto A., Ishizaki S., Shiomi K. (2009). Tropomyosins in Gastropods and Bivalves: Identification as Major Allergens and Amino Acid Sequence Features. Food Chem..

[26] Giannetti A., Pession A., Bettini I., Ricci G., Giannì G., Caffarelli C. (2023). IgE Mediated Shellfish Allergy in Children—A Review. Nutrients.

[27] Vidal C., Bartolomé B., Rodríguez V., Armisén M., Linneberg A., González-Quintela A. (2015). Sensitization Pattern of Crustacean-Allergic Individuals Can Indicate Allergy to Molluscs. Allergy.

[28] Ivanović J., Baltić M.Ž., Bošković M., Kilibarda N., Dokmanović M., Marković R., Janjić J., Baltić B. (2017). Anisakis Allergy in Human. Trends Food Sci. Technol..

[29] Traidl-Hoffmann C., Zuberbier T., Werfel T. (2022). Allergic Diseases—From Basic Mechanisms to Comprehensive Management and Prevention.

[30] Hazebrouck S., Awad Y., Bernard H. (2025). Nouvelles Sources Protéiques et Nouveaux Allergènes: Sensibilisation de novo et Réactivité Croisée. Rev. Française D’allergologie.

[31] Awad Y., Bernard H., Adel-Patient K., Hazebrouck S. (2025). New Dietary Trends and Alternative Proteins: The Emergence of Novel Food Allergens. Curr. Opin. Clin. Nutr. Metab. Care.

[32] Calvin K., Dasgupta D., Krinner G., Mukherji A., Thorne P.W., Trisos C., Romero J., Aldunce P., Barrett K., Blanco G., Lee H., Romero J. (2023). IPCC, 2023: Climate Change 2023: Synthesis Report. Contribution of Working Groups I, II and III to the Sixth Assessment Report of the Intergovernmental Panel on Climate Change.

[33] Poloczanska E.S., Burrows M.T., Brown C.J., García Molinos J., Halpern B.S., Hoegh-Guldberg O., Kappel C.V., Moore P.J., Richardson A.J., Schoeman D.S. (2016). Responses of Marine Organisms to Climate Change across Oceans. Front. Mar. Sci..

[34] Bottacini D., Pollux B.J.A., Nijland R., Jansen P.A., Naguib M., Kotrschal A. (2024). Lionfish (*Pterois miles*) in the Mediterranean Sea: A Review of the Available Knowledge with an Update on the Invasion Front. NeoBiota.

[35] Asea A.A.A., Kaur P. (2018). Regulation of Heat Shock Protein Responses.

[36] Liebler D.C. (2002). Introduction to Proteomics: Tools for the New Biology.

[37] Keshavarz B., Jiang X., Hsieh Y.-H.P., Rao Q. (2019). Matrix Effect on Food Allergen Detection—A Case Study of Fish Parvalbumin. Food Chem..

[38] Nugraha R., Ruethers T., Johnston E.B., Rolland J.M., O’Hehir R.E., Kamath S.D., Lopata A.L. (2021). Effects of Extraction Buffer on the Solubility and Immunoreactivity of the Pacific Oyster Allergens. Foods.

[39] Zhao J., Li Y., Xu L., Timira V., Zhang Z., Chen G., Zhang L., Lin H., Li Z. (2022). Improved Protein Extraction from Thermally Processed Shrimp (*Litopenaeus vannamei*) for Reliable Immunodetection via a Synergistic Effect of Buffer Additives. LWT.

[40] Faisal M., Vasiljevic T., Donkor O.N. (2019). A Review on Methodologies for Extraction, Identification and Quantification of Allergenic Proteins in Prawns. Food Res. Int..

[41] Fan S., Ma J., Li C., Wang Y., Zeng W., Li Q., Zhou J., Wang L., Wang Y., Zhang Y. (2022). Determination of Tropomyosin in Shrimp and Crab by Liquid Chromatography–Tandem Mass Spectrometry Based on Immunoaffinity Purification. Front. Nutr..

[42] Anđelković U., Martinović T., Josić D. (2015). Foodomic Investigations of Food Allergies. Curr. Opin. Food Sci..

[43] Ruethers T., Taki A.C., Khangurha J., Roberts J., Buddhadasa S., Clarke D., Hedges C.E., Campbell D.E., Kamath S.D., Lopata A.L. (2020). Commercial Fish ELISA Kits Have a Limited Capacity to Detect Different Fish Species and Their Products. J. Sci. Food Agric..

[44] Carrera M., Abril A.G., Pazos M., Calo-Mata P., Villa T.G., Barros-Velázquez J. (2024). Proteins and Peptides: Proteomics Approaches for Food Authentication and Allergen Profiling. Curr. Opin. Food Sci..

[45] Koeberl M., Clarke D., Lopata A.L. (2014). Next Generation of Food Allergen Quantification Using Mass Spectrometric Systems. J. Proteome Res..

[46] Zhang Y., Fonslow B.R., Shan B., Baek M.-C., Yates J.R.I. (2013). Protein Analysis by Shotgun/Bottom-up Proteomics. Chem. Rev..

[47] Miller R.M., Smith L.M. (2023). Overview and Considerations in Bottom-up Proteomics. Analyst.

[48] Radauer C., Breiteneder H. (2019). Allergen Databases—A Critical Evaluation. Allergy.

[49] Borràs E., Sabidó E. (2017). What Is Targeted Proteomics? A Concise Revision of Targeted Acquisition and Targeted Data Analysis in Mass Spectrometry. Proteomics.

[50] Aebersold R., Bensimon A., Collins B.C., Ludwig C., Sabido E. (2016). Applications and Developments in Targeted Proteomics: From SRM to DIA/SWATH. Proteomics.

[51] Peterson A.C., Russell J.D., Bailey D.J., Westphall M.S., Coon J.J. (2012). Parallel Reaction Monitoring for High Resolution and High Mass Accuracy Quantitative, Targeted Proteomics. Mol. Cell Proteom..

[52] Zhao L., Cong X., Zhai L., Hu H., Xu J.-Y., Zhao W., Zhu M., Tan M., Ye B.-C. (2020). Comparative Evaluation of Label-Free Quantification Strategies. J. Proteom..

[53] Kani K., Comai L., Katz J.E., Mallick P. (2017). Quantitative Proteomics Using SILAC. Proteomics: Methods and Protocols.

[54] Zhang L., Elias J.E. (2017). Relative Protein Quantification Using Tandem Mass Tag Mass Spectrometry. Methods Mol. Biol..

[55] Kettenbach A.N., Rush J., Gerber S.A. (2011). Absolute Quantification of Protein and Post-Translational Modification Abundance with Stable Isotope–Labeled Synthetic Peptides. Nat. Protoc..

[56] Olsen J.V., Mann M. (2004). Improved Peptide Identification in Proteomics by Two Consecutive Stages of Mass Spectrometric Fragmentation. Proc. Natl. Acad. Sci. USA.

[57] Yates J.R., Kelleher N.L. (2013). Top Down Proteomics. Anal. Chem..

[58] Kuehn A., Swoboda I., Arumugam K., Hilger C., Hentges F. (2014). Fish Allergens at a Glance: Variable Allergenicity of Parvalbumins, the Major Fish Allergens. Front. Immunol..

[59] Perez-Gordo M., Lin J., Bardina L., Pastor-Vargas C., Cases B., Vivanco F., Cuesta-Herranz J., Sampson H.A. (2012). Epitope Mapping of Atlantic Salmon Major Allergen by Peptide Microarray Immunoassay. Int. Arch. Allergy Immunol..

[60] Brodbelt J.S., Morrison L.J., Santos I. (2020). Ultraviolet Photodissociation Mass Spectrometry for Analysis of Biological Molecules. Chem. Rev..

[61] Carrera M., Weisbrod C., Lopez-Ferrer D., Huguet R., Gallardo J.M., Schwartz J., Huhmer A. (2015). Top-Down, High-throughput of Thermo-Stable Allergens Using Complementary MS/MS Fragmentation Strategies.

[62] Remington B.C., Westerhout J., Meima M.Y., Blom W.M., Kruizinga A.G., Wheeler M.W., Taylor S.L., Houben G.F., Baumert J.L. (2020). Updated Population Minimal Eliciting Dose Distributions for Use in Risk Assessment of 14 Priority Food Allergens. Food Chem. Toxicol..

[63] Westerhout J., Baumert J.L., Blom W.M., Allen K.J., Ballmer-Weber B., Crevel R.W.R., Dubois A.E.J., Fernández-Rivas M., Greenhawt M.J., Hourihane J.O. (2019). Deriving Individual Threshold Doses from Clinical Food Challenge Data for Population Risk Assessment of Food Allergens. J. Allergy Clin. Immunol..

[64] VITAL® Voluntary Incidental Trace Allergen Labelling. https://vital.allergenbureau.net/.

[65] Taylor S.B., Christensen G., Grinter K., Sherlock R., Warren L. (2018). The Allergen Bureau VITAL Program. J. AOAC Int..

[66] Holzhauser T., Johnson P., Hindley J.P., O’Connor G., Chan C.-H., Costa J., Fæste C.K., Hirst B.J., Lambertini F., Miani M. (2020). Are Current Analytical Methods Suitable to Verify VITAL^®^ 2.0/3.0 Allergen Reference Doses for EU Allergens in Foods?. Food Chem. Toxicol..

[67] Wilm M. (2009). Quantitative Proteomics in Biological Research. Proteomics.

[68] Hsu J.-L., Huang S.-Y., Chow N.-H., Chen S.-H. (2003). Stable-Isotope Dimethyl Labeling for Quantitative Proteomics. Anal. Chem..

[69] Burkhart J.M., Vaudel M., Zahedi R.P., Martens L., Sickmann A. (2011). iTRAQ Protein Quantification: A Quality-Controlled Workflow. Proteomics.

[70] Gavage M., Van Vlierberghe K., Van Poucke C., De Loose M., Gevaert K., Dieu M., Renard P., Arnould T., Filee P., Gillard N. (2020). Comparative Study of Concatemer Efficiency as an Isotope-Labelled Internal Standard for Allergen Quantification. Food Chem..

[71] Kandi S., Savaryn J.P., Ji Q.C., Jenkins G.J. (2022). Use of In-Sample Calibration Curve Approach for Quantification of Peptides with High-Resolution Mass Spectrometry. Rapid Commun. Mass Spectrom..

[72] Hernández-Mesa M., Ropartz D., García-Campaña A.M., Rogniaux H., Dervilly-Pinel G., Le Bizec B. (2019). Ion Mobility Spectrometry in Food Analysis: Principles, Current Applications and Future Trends. Molecules.

[73] Carrera M. (2020). Proteómica y biología de sistemas para el estudio de la alergia alimentaria. Arbor.

[74] Kokesch-Himmelreich J., Wittek O., Race A.M., Rakete S., Schlicht C., Busch U., Römpp A. (2022). MALDI Mass Spectrometry Imaging: From Constituents in Fresh Food to Ingredients, Contaminants and Additives in Processed Food. Food Chem..

[75] Szklarczyk D., Kirsch R., Koutrouli M., Nastou K., Mehryary F., Hachilif R., Gable A.L., Fang T., Doncheva N.T., Pyysalo S. (2023). The STRING Database in 2023: Protein-Protein Association Networks and Functional Enrichment Analyses for Any Sequenced Genome of Interest. Nucleic Acids Res..

[76] Shannon P., Markiel A., Ozier O., Baliga N.S., Wang J.T., Ramage D., Amin N., Schwikowski B., Ideker T. (2003). Cytoscape: A Software Environment for Integrated Models of Biomolecular Interaction Networks. Genome Res..

[77] Beck A.G., Muhoberac M., Randolph C.E., Beveridge C.H., Wijewardhane P.R., Kenttämaa H.I., Chopra G. (2024). Recent Developments in Machine Learning for Mass Spectrometry. ACS Meas. Sci. Au.

[78] Gessulat S., Schmidt T., Zolg D.P., Samaras P., Schnatbaum K., Zerweck J., Knaute T., Rechenberger J., Delanghe B., Huhmer A. (2019). Prosit: Proteome-Wide Prediction of Peptide Tandem Mass Spectra by Deep Learning. Nat. Methods.

[79] Frejno M., Berger M.T., Tüshaus J., Hogrebe A., Seefried F., Graber M., Samaras P., Ben Fredj S., Sukumar V., Eljagh L. (2025). Unifying the Analysis of Bottom-up Proteomics Data with CHIMERYS. Nat. Methods.

[80] Carrera M., Cañas B., Gallardo J.M. (2012). Rapid Direct Detection of the Major Fish Allergen, Parvalbumin, by Selected MS/MS Ion Monitoring Mass Spectrometry. J. Proteom..

[81] Sun L., Lin H., Li Z., Sun W., Wang J., Wu H., Ge M., Ahmed I., Pavase T.R. (2019). Development of a Method for the Quantification of Fish Major Allergen Parvalbumin in Food Matrix via Liquid Chromatography-Tandem Mass Spectrometry with Multiple Reaction Monitoring. Food Chem..

[82] Piovesana S., Capriotti A.L., Caruso G., Cavaliere C., La Barbera G., Zenezini Chiozzi R., Laganà A. (2016). Labeling and Label Free Shotgun Proteomics Approaches to Characterize Muscle Tissue from Farmed and Wild Gilthead Sea Bream (*Sparus aurata*). J. Chromatogr. A.

[83] Westfalewicz B., Dietrich M.A., Irnazarow I., Ciereszko A. (2015). Identification of 5–15 kDa Substances in Carp Seminal Plasma Using Mass Spectrometry. J. Appl. Ichthyol..

[84] Ruethers T., Taki A.C., Nugraha R., Cao T.T., Koeberl M., Kamath S.D., Williamson N.A., O’Callaghan S., Nie S., Mehr S.S. (2019). Variability of Allergens in Commercial Fish Extracts for Skin Prick Testing. Allergy.

[85] Liu Q., Sui Z., Feng N., Huang Y., Li Y., Ahmed I., Ruethers T., Liang H., Li Z., Lopata A.L. (2024). Characterization, Epitope Confirmation, and Cross-Reactivity Analysis of Parvalbumin from *Lateolabrax maculatus* by Multiomics Technologies. J. Agric. Food Chem..

[86] Carrera M., Cañas B., Vázquez J., Gallardo J.M. (2010). Extensive de novo Sequencing of New Parvalbumin Isoforms Using a Novel Combination of Bottom-Up Proteomics, Accurate Molecular Mass Measurement by FTICR−MS, and Selected MS/MS Ion Monitoring. J. Proteome Res..

[87] Aiello D., Materazzi S., Risoluti R., Thangavel H., Di Donna L., Mazzotti F., Casadonte F., Siciliano C., Sindona G., Napoli A. (2015). A Major Allergen in Rainbow Trout (*Oncorhynchus mykiss*): Complete Sequences of Parvalbumin by MALDI Tandem Mass Spectrometry. Mol. BioSyst..

[88] Zhao X., Lu J., Long S., Soko W.C., Qin Q., Qiao L., Bi H. (2021). MALDI-TOF MS and Magnetic Beads for Rapid Seafood Allergen Tests. J. Agric. Food Chem..

[89] Zhao X., Bi H. (2025). Impact of Boiling on the Allergens in Fish Bone Samples Identified by Microfluidic Chips and MALDI-TOF MS. Food Chem..

[90] Lin F., Soko W.C., Xie J., Bi H. (2023). On-Chip Discovery of Allergens from the Exudate of Large Yellow Croaker (*Larimichthys crocea*) Muscle Food by Matrix-Assisted Laser Desorption/Ionization Time-of-Flight Mass Spectrometry. J. Agric. Food Chem..

[91] Carrera M., Fidalgo L.G., Saraiva J.A., Aubourg S.P. (2018). Effects of High-Pressure Treatment on the Muscle Proteome of Hake by Bottom-Up Proteomics. J. Agric. Food Chem..

[92] Wu Y., Yao K., Yang Y., Wu X., Zhang J., Jin Y., Xing Y., Niu Y., Jiang Q., Dai C. (2024). A SILAC-Based Accurate Quantification of Shrimp Allergen Tropomyosin in Complex Food Matrices Using UPLC-MS/MS. Food Chem..

[93] Ho C.-W., Hsu J.-L., Chen S.-H., Liaw E.-T., Liu S.-S., Huang E.S., Chen Y.-K., Jean Huang C.-C., Yu H.-S. (2021). Development and Validation of Mass Spectrometry-Based Method for Detecting Shrimp Allergen Tropomyosin. LWT.

[94] Lu Y., Zhang H., Gao H., Zhang X., Ji H., Gao C., Chen Y., Xiao J., Li Z. (2024). Quantification of Allergic Crustacean Tropomyosin Using Shared Signature Peptides in Processed Foods with a Mass Spectrometry-Based Proteomic Strategy. J. Agric. Food Chem..

[95] Yao K., Yang Y., Liu J., Zhang J., Shao B., Zhang Y. (2021). Labeled Peptide-Free UHPLC–MS/MS Method Used for Simultaneous Determination of Shrimp and Soybean in Sauce Products. J. Agric. Food Chem..

[96] Korte R., Monneuse J.-M., Gemrot E., Metton I., Humpf H.-U., Brockmeyer J. (2016). New High-Performance Liquid Chromatography Coupled Mass Spectrometry Method for the Detection of Lobster and Shrimp Allergens in Food Samples via Multiple Reaction Monitoring and Multiple Reaction Monitoring Cubed. J. Agric. Food Chem..

[97] Srisomsap C., Nonthawong K., Chokchaichamnankit D., Svasti J., Phiriyangkul P. (2023). Shotgun Proteomics Characterization of Potential Allergens in Dried and Powdered Krill and Fresh and Powdered Whiteleg Shrimp. Food Biosci..

[98] Nugraha R., Kamath S.D., Johnston E., Zenger K.R., Rolland J.M., O’Hehir R.E., Lopata A.L. (2018). Rapid and Comprehensive Discovery of Unreported Shellfish Allergens Using Large-Scale Transcriptomic and Proteomic Resources. J. Allergy Clin. Immunol..

[99] Rolland J.M., Varese N.P., Abramovitch J.B., Anania J., Nugraha R., Kamath S., Hazard A., Lopata A.L., O’Hehir R.E. (2018). Effect of Heat Processing on IgE Reactivity and Cross-Reactivity of Tropomyosin and Other Allergens of Asia-Pacific Mollusc Species: Identification of Novel Sydney Rock Oyster Tropomyosin Sac g 1. Mol. Nutr. Food Res..

[100] Liu M., Han T.-J., Huan F., Li M.-S., Xia F., Yang Y., Wu Y.-H., Chen G.-X., Cao M.-J., Liu G.-M. (2021). Effects of Thermal Processing on the Allergenicity, Structure, and Critical Epitope Amino Acids of Crab Tropomyosin. Food Funct..

[101] Carrera M., Gallardo J.M., Pascual S., González Á.F., Medina I. (2016). Protein Biomarker Discovery and Fast Monitoring for the Identification and Detection of Anisakids by Parallel Reaction Monitoring (PRM) Mass Spectrometry. J. Proteom..

[102] Fæste C.K., Moen A., Schniedewind B., Haug Anonsen J., Klawitter J., Christians U. (2016). Development of Liquid Chromatography-Tandem Mass Spectrometry Methods for the Quantitation of Anisakis Simplex Proteins in Fish. J. Chromatogr. A.

[103] Fæste C.K., Jonscher K.R., Dooper M.M.W.B., Egge-Jacobsen W., Moen A., Daschner A., Egaas E., Christians U. (2014). Characterisation of Potential Novel Allergens in the Fish Parasite Anisakis Simplex. EuPA Open Proteom..

[104] Ma Y., Griesmeier U., Susani M., Radauer C., Briza P., Erler A., Bublin M., Alessandri S., Himly M., Vàzquez-Cortés S. (2008). Comparison of Natural and Recombinant Forms of the Major Fish Allergen Parvalbumin from Cod and Carp. Mol. Nutr. Food Res..

[105] Huang M.-C., Ochiai Y. (2005). Fish Fast Skeletal Muscle Tropomyosins Show Species-Specific Thermal Stability. Comp. Biochem. Physiol. Part B Biochem. Mol. Biol..

[106] Dasanayaka B.P., Li Z., Pramod S.N., Chen Y., Khan M.U., Lin H. (2022). A Review on Food Processing and Preparation Methods for Altering Fish Allergenicity. Crit. Rev. Food Sci. Nutr..

[107] Pi X., Zhu L., Liu J., Zhang B. (2024). Effect of Thermal Processing on Food Allergenicity: Mechanisms, Application, Influence Factor, and Future Perspective. J. Agric. Food Chem..

[108] Taki A.C., Ruethers T., Nugraha R., Karnaneedi S., Williamson N.A., Nie S., Leeming M.G., Mehr S.S., Campbell D.E., Lopata A.L. (2023). Thermostable Allergens in Canned Fish: Evaluating Risks for Fish Allergy. Allergy.

[109] Schrama D., Raposo De Magalhães C., Cerqueira M., Carrilho R., Revets D., Kuehn A., Engrola S., Rodrigues P.M. (2022). Fish Processing and Digestion Affect Parvalbumins Detectability in Gilthead Seabream and European Seabass. Animals.

[110] Chen B., He H., Wang X., Wu S., Wang Q., Zhang J., Qiao Y., Liu H. (2025). Research Progress on Shrimp Allergens and Allergenicity Reduction Methods. Foods.

[111] Khan M.U., Ahmed I., Lin H., Li Z., Costa J., Mafra I., Chen Y., Wu Y.-N. (2019). Potential Efficacy of Processing Technologies for Mitigating Crustacean Allergenicity. Crit. Rev. Food Sci. Nutr..

[112] Birse N., Burns D.T., Walker M.J., Quaglia M., Elliott C.T. (2023). Food Allergen Analysis: A Review of Current Gaps and the Potential to Fill Them by Matrix-assisted Laser Desorption/Ionization. Compr. Rev. Food Sci. Food Saf..

[113] Guergues J., Wohlfahrt J., Stevens S.M. (2022). Enhancement of Proteome Coverage by Ion Mobility Fractionation Coupled to PASEF on a TIMS-QTOF Instrument. J. Proteome Res..

[114] Krieger J.R., Wybenga-Groot L.E., Tong J., Bache N., Tsao M.S., Moran M.F. (2019). Evosep One Enables Robust Deep Proteome Coverage Using Tandem Mass Tags While Significantly Reducing Instrument Time. J. Proteome Res..

[115] Jafari S., Guercetti J., Geballa-Koukoula A., Tsagkaris A.S., Nelis J.L.D., Marco M.-P., Salvador J.-P., Gerssen A., Hajslova J., Elliott C. (2021). ASSURED Point-of-Need Food Safety Screening: A Critical Assessment of Portable Food Analyzers. Foods.

[116] Planque M., Arnould T., Renard P., Delahaut P., Dieu M., Gillard N. (2017). Highlight on Bottlenecks in Food Allergen Analysis: Detection and Quantification by Mass Spectrometry. J. AOAC Int..

[117] Pedreschi R., Nørgaard J., Maquet A. (2012). Current Challenges in Detecting Food Allergens by Shotgun and Targeted Proteomic Approaches: A Case Study on Traces of Peanut Allergens in Baked Cookies. Nutrients.

[118] Paez V., Barrett W.B., Deng X., Diaz-Amigo C., Fiedler K., Fuerer C., Hostetler G.L., Johnson P., Joseph G., Konings E.J.M. (2016). AOAC SMPR^®^ 2016.002. J. AOAC Int..

[119] Planque M., Arnould T., Dieu M., Delahaut P., Renard P., Gillard N. (2017). Liquid Chromatography Coupled to Tandem Mass Spectrometry for Detecting Ten Allergens in Complex and Incurred Foodstuffs. J. Chromatogr. A.

[120] Ogura T., Clifford R., Oppermann U. (2019). Simultaneous Detection of 13 Allergens in Thermally Processed Food Using Targeted LC–MS/MS Approach. J. AOAC Int..

[121] Abdel Rahman A.M., Lopata A.L., O’Hehir R.E., Robinson J.J., Banoub J.H., Helleur R.J. (2010). Characterization and de novo Sequencing of Snow Crab Tropomyosin Enzymatic Peptides by Both Electrospary Ionization and Matrix-assisted Laser Desorption Ionization QqToF Tandem Mass Spectrometry. J. Mass Spectrom..

[122] Mykles D.L., Cotton J.L.S., Taniguchi H. (1998). Cloning of Tropomyosins from Lobster (*Homarus americanus*) Striated Muscles: Fast and Slow Isoforms May Be Generated from the Same Transcript. J. Muscle Res. Cell Motil..

[123] Shin J.H., Reddy Y.V.M., Park T.J., Park J.P. (2022). Recent Advances in Analytical Strategies and Microsystems for Food Allergen Detection. Food Chem..

[124] Rocha J.P., Freitas M., Geraldo D., Bento F., Delerue-Matos C., Nouws H.P.A. (2024). Electrochemical Magnetic Immunoassay for the Determination of the Fish Allergen β-Parvalbumin. Biosensors.

[125] Jiang D., Xu Y., Jiang H., Xiang X., Wang L. (2025). A Biomimetic Skin Microtissue Biosensor for the Detection of Fish Parvalbumin. Bioelectrochemistry.

[126] Chinnappan R., Rahamn A.A., AlZabn R., Kamath S., Lopata A.L., Abu-Salah K.M., Zourob M. (2020). Aptameric Biosensor for the Sensitive Detection of Major Shrimp Allergen, Tropomyosin. Food Chem..

[127] Amouzadeh Tabrizi M., Shamsipur M., Saber R., Sarkar S., Ebrahimi V. (2017). A High Sensitive Visible Light-Driven Photoelectrochemical Aptasensor for Shrimp Allergen Tropomyosin Detection Using Graphitic Carbon Nitride-TiO_2_ Nanocomposite. Biosens. Bioelectron..

[128] Zhou J., Wang Y., Zheng L., Li H. (2025). An Aptamer-Initiated Catalytic Hairpin Assembly Fluorescent Biosensor for Simultaneous Detection of Major Seafood Allergens in Food System. Microchem. J..

[129] Cook Q.S., Burks A.W. (2018). Peptide and Recombinant Allergen Vaccines for Food Allergy. Clin. Rev. Allergy Immunol..

[130] Swoboda I., Balic N., Klug C., Focke M., Weber M., Spitzauer S., Neubauer A., Quirce S., Douladiris N., Papadopoulos N.G. (2013). A General Strategy for the Generation of Hypoallergenic Molecules for the Immunotherapy of Fish Allergy. J. Allergy Clin. Immunol..

[131] Freidl R., Gstoettner A., Baranyi U., Swoboda I., Stolz F., Focke-Tejkl M., Wekerle T., Van Ree R., Valenta R., Linhart B. (2017). Blocking Antibodies Induced by Immunization with a Hypoallergenic Parvalbumin Mutant Reduce Allergic Symptoms in a Mouse Model of Fish Allergy. J. Allergy Clin. Immunol..

[132] Zhang J., Liu W., Zhang R., Zhao X., Fang L., Qin X., Gu R., Lu J., Li G. (2020). Hypoallergenic Mutants of the Major Oyster Allergen Cra g 1 Alleviate Oyster Tropomyosin Allergenic Potency. Int. J. Biol. Macromol..

[133] Huan F., Gao S., Ni L.-N., Wu M.-X., Gu Y., Yun X., Liu M., Lai D., Xiao A.-F., Liu G.-M. (2024). Development of Hypoallergenic Derivatives of Cra a 1 with B Cell Epitope Deletion and T Cell Epitope Retention. J. Agric. Food Chem..

